# Primary LYmphedema Multidisciplinary Approach in Patients Affected by Primary Lower Extremity Lymphedema

**DOI:** 10.3390/jcm13175161

**Published:** 2024-08-30

**Authors:** Pedro Ciudad, Alberto Bolletta, Juste Kaciulyte, Oscar J. Manrique, Joseph M. Escandón

**Affiliations:** 1 Department of Plastic, Reconstructive and Burn Surgery, Arzobispo Loayza National Hospital, Lima 15082, Peru; oscarj.manrique@gmail.com; 2 Academic Department of Surgery, Faculty of Medicine, Federico Villarreal National University, Lima 15084, Peru; 3 Plastic Surgery Unit, Department of Translational Research and New Technologies in Medicine and Surgery, University of Pisa, 56125 Pisa, Italy; alb.bolletta@gmail.com; 4 Unit of Plastic and Reconstructive Surgery, Department of Surgery “P.Valdoni”, Sapienza University of Rome, Policlinico Umberto I, 00185 Rome, Italy; justekc@gmail.com; 5 Division of Plastic and Reconstructive Surgery, Strong Memorial Hospital, University of Rochester Medical Center, Rochester, NY 14642, USA; 6 Department of Surgery, Mayo Clinic, Rochester, MN 55905, USA; joseph.escandon.medical@gmail.com

**Keywords:** lymphedema, supermicrosurgery, suction-assisted lipectomy (SAL), lymphatico-venous anastomoses (LVA), vascularized lymph node transfer

## Abstract

**Background**: Primary lymphedema is a chronic condition caused by a developmental abnormality of the lymphatic system, leading to its malfunction. Various surgical options, including physiologic and excisional procedures, have been proposed. The aim of this study was to present a comprehensive algorithm for the treatment of primary lower extremity lymphedema: the Primary LYmphedema Multidisciplinary Approach (P-LYMA). **Methods**: Nineteen patients were treated following the P-LYMA protocol. Patients underwent pre- and postoperative complex decongestive therapy (CDT). A variety of physiologic and excisional procedures were performed, either independently or in combination. The primary outcome was to assess the circumferential reduction rate (CRR). The Lymphedema Quality of Life Score (LeQOLiS), reduction in the number of cellulitis episodes, and complications were recorded. **Results**: The mean CRR was 73 ± 20% at twelve months postoperatively. The frequency of cellulitis episodes per year decreased from a mean of 1.9 ± 0.8 preoperatively to 0.4 ± 0.6 during follow-up. Two patients experienced minor complications. The mean hospitalization time was 5 days. Patients’ quality of life, as measured by the LeQOLiS, significantly improved from 70.4 ± 12 preoperatively to 24 ± 14 at twelve months postoperatively. **Conclusions**: The P-LYMA algorithm maximizes surgical outcomes and improves the quality of life in patients with primary lymphedema. CDT is essential for optimizing results.

## 1. Introduction

Primary lymphedema is a chronic condition caused by a developmental abnormality of the lymphatic system and its malfunction [1]. Primary lymphedema is considered rare, and the estimated worldwide prevalence is about 1 in 100,000 individuals. It can manifest as an isolated disease or as part of a complex syndrome [2]. Although primary lymphedema usually presents in childhood, it may occur at any age [2]. The natural history of the disease is variable, and it depends not only on the underlying developmental abnormality but also on the penetrance and expression of the genetic condition. In almost 30% of primary lymphedema patients, a genetic mutation is identifiable, and it often involves the signaling pathway of vascular endothelial growth factor C [3]. More than 20 genes have been related to lymphatic abnormalities in primary lymphedema with a high degree of genetic heterogeneity [3].

Primary lymphedema usually involves the lower extremities, but on rare occasions, it can affect the genitalia and upper limbs. In general, this condition affects females twice as much as males [4]. The phenotypic entities of primary lymphedema vary in age of onset, site of edema, associated features, inheritance patterns, and underlying genetic cause [4,5]. Depending on the time of onset, primary lymphedema can be classified into three types: congenital lymphedema, lymphedema praecox, and lymphedema tarda [5]. Congenital lymphedema presents at birth or in the first 2 years of life, lymphedema praecox typically presents during puberty or before the age of 35, and lymphedema tarda appears after the age of thirty-five [5].

The pathogenesis of most lymphatic disorders is not fully understood, and it is an evolving area of research [1]. According to Papendieck et al., primary lymphedema can be classified into three groups depending on the histologic and anatomical components that are affected by the developmental abnormality [6]. It can be related to interstitial lymphatic endothelial dysplasia, lymphangiodysplasia, or lymphadenodysplasia [6]. This classification provides the basis for a physiologic approach to primary lymphedema and dictates how to provide a comprehensive treatment taking into consideration the needs of patients.

Different physiologic procedures have been proposed in lymphatic surgery with the aim of restoring lymphatic drainage. Lymphaticovenous anastomosis (LVA) is a surgical approach design to divert lymph into the venous system, bypassing areas of damaged or obstructed lymphatics vessels [7]. On the other hand, vascularized lymph node transfer (VLNT) involves the auto-transplantation of functional lymph nodes to restore physiologic lymphatic flow in a lymphedematous limb [8,9,10]. In advanced stages, lymph-stasis and chronic inflammation cause alterations of the subcutaneous tissue, resulting in adipogenesis and secondary fibrosis. In these cases, excisional procedures are required to enhance the results of physiologic procedures. By improving the lymphatic drainage and eliminating the densely fibrotic skin and subcutaneous tissue, the combination of physiologic and excisional procedures can address both pathophysiological components simultaneously [11,12].

The aim of this work is to present the Primary-LYmphedema Multidisciplinary Approach (P-LYMA), a complete algorithm for the management of primary lymphedema based on a combination of conservative and surgical therapies. Furthermore, we meticulously described how physiologic and excisional procedures were chosen and performed to address the fluid and solid components of primary lymphedema.

## 2. Materials and Methods

After approval by the Institutional Review Board, patients presenting to a large academic medical center in Lima, Peru, with a diagnosis of primary lymphedema of the lower limbs were enrolled in this prospective study. Initially, patients were evaluated in an outpatient setting and underwent further imaging tests to assess for possible lymphedema following a clinical examination that raised concerns for primary lymphedema. Exclusion criteria included concomitant vascular malformations related to the development of lymphedema, previous operations to the affected limb, or trauma involving the lower limbs. Patients with secondary lymphedema or patients who were not compliant with our algorithm were excluded from this study.

### 2.1. Study Protocol

**Figure 1:**
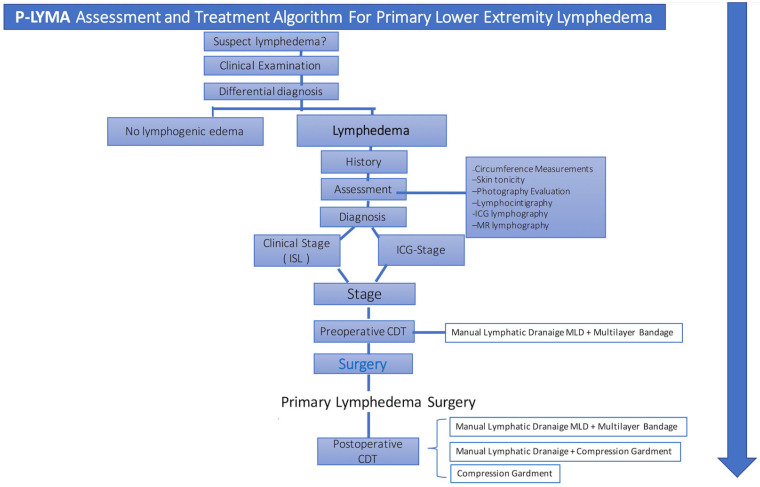
Assessment and treatment algorithm. Primary-Lymphedema Multidisciplinary Approach (P-LYMA) for patients with lower extremity lymphedema. CDT: complete decongestive therapy, ISL: International Society of Lymphology, ICG: indocyanine green.

The diagnosis of primary lymphedema was performed by means of the medical history and clinical evaluation. Further confirmation was determined by performing lymphoscintigraphy and ICG lymphography (Figure 1). Additional data such as duration of symptoms, prior episodes of infection, and previous conservative treatment were recorded. Circumferential limb and body weight measurements together with preoperative quality of life assessment were recorded. Circumferential measurements were taken at four levels: midfoot, ankle (at the level of the medial and lateral malleoli), 10 cm below the knee, and 10 cm above the knee.

**Figure 2:**
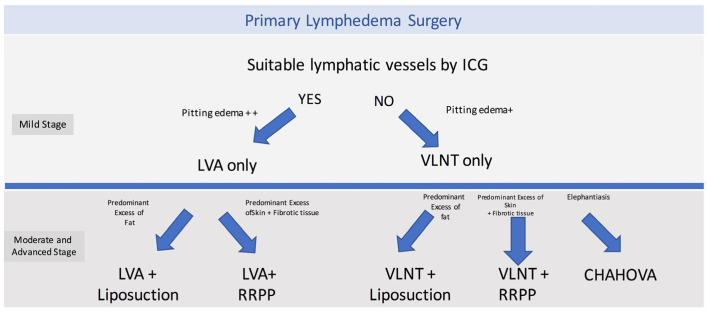
P-LYMA surgical algorithm for primary lymphedema. LVA: lymphaticovenous anastomosis, VLNT: vascularized lymph node transfer, RRRP: radical reduction with preservation of perforators, CHAHOVA: Charles’, Homans’, and vascularized lymph node transfer procedures. Stage ISL I or IIA was regarded as mild stages, while ISL IIB or III was considered moderate or advanced stages. The decision to pursue a resective procedure in stage ISL IIA included a comprehensive evaluation of duration, severity, and progression of symptoms.

Lymphoscintigraphy was performed on patients preoperatively to assess lymphatic drainage from lower limbs. All patients were staged according to the International Society of Lymphology (ISL) system. Patients were also evaluated and stratified based on ICG lymphography. ICG was injected at the second and fourth web space of the foot, the medial and lateral border of the Achilles’ tendon, the lateral and medial aspect of the knee, and at the groin. Prior to surgical treatment, all patients underwent physiotherapy with complete decongestive therapy (CDT) for 6 months which consisted of manual lymphatic drainage, multilayer bandage compression therapy, remedial exercises, and skin care [13]. Clinical assessment and imaging were performed before starting CDT (Figure 2). Postoperatively, patients received CDT too.

### 2.2. Treatment Algorithm

The surgical treatment options included physiologic procedures such as LVA or VLNT, and excisional procedures such as liposuction, radical reduction with preservation of perforators (RRPP), or the combined Charles’, Homan’s, and VLNT (CHAHOVA). The choice of the procedure depended on preoperative evaluation and clinical staging. LVA was performed when ICG lymphography showed areas of linear or splash patterns that were interrupted by areas of dermal backflow. Depending on the number of suitable vessels found during preoperative ICG lymphography, two or three LVAs were performed per extremity. On the other hand, if suitable lymphatics were not found on ICG lymphography, a gastroepiploic or supraclavicular VLNT was planned.

Depending on staging and clinical evaluation, patients were selected to receive additional excisional procedures. Patients with ISL stage IIB were treated with liposuction if they presented with predominant adipose tissue deposition. On the other hand, in stage III patients who presented with highly fibrotic subcutaneous tissue and recurrent skin infections, a more invasive procedure was performed (RRPP or CHAHOVA). RRPP was chosen in patients with extensive skin excess and good skin quality [14,15]. Conversely, the combination of Charles’ and Homan’s procedure (CHAHOVA) was used for en bloc resection of the skin and subcutaneous tissue, and it was performed in patients with damaged skin and indurated/fibrotic subcutaneous tissues [14,15].

Patients started CDT 5 to 24 days after surgery depending on the surgical procedure. Patients who underwent liposuction + LVA started CDT 5 days after surgery, as early CDT enhances LVA function. Those who received liposuction + VLNT or RRPP + VLNT started CDT 14 days after surgery to allow the lymph node flap to adapt to new hemodynamic changes and avoid surgical site wound disruption. Those who received the CHAHOVA approach started CDT 21–24 days after surgery to allow skin grafts to take, avoid bleeding, and avoid traumatic injury to the reconstruction. CDT was initially performed with continuous multilayer bandage compression therapy and sessions of manual lymphatic drainage for the first three months, three times per week.

Circumferential reduction rates were evaluated monthly during these first three months. The continuous multilayer bandage compression therapy was then transitioned into compression garments. The number of weekly sessions of manual lymphatic drainage was progressively reduced over the following three months and completed by six months after surgery.

### 2.3. Outcomes

Circumferential reduction rates (CRRs) were recorded one month and two months after surgery. After this time, circumferential reduction rates were evaluated together alongside the Lymphedema Quality of Life Score (LeQOLiS) at 3, 6, 12, and 24 months after surgery. The CRR was defined as the difference in percentage, between the affected limb (AL) circumference and the non-affected limb (NAL) circumference, as determined with the following equation: circumference reduction rate (%) = (1 − [postoperative AL − NAL]/[preoperative AL − NAL]) × 100. Due to the symmetrical component of bilateral lower limb lymphedema, circumferential reduction rates were only calculated for unilateral cases. Additionally, we recorded and analyzed the number of pre- and postoperative episodes of cellulitis, length of stay, complications, and pre- and postoperative LeQOLiS.

## 3. Results

Nineteen patients with primary lower extremity lymphedema were included, three patients with bilateral limb compromise, and sixteen with unilateral limb compromise. Eleven patients were male (58%) and eight were female (42%). The mean age of patients at diagnosis was 29 ± 11 years, while the mean BMI was 29 ± 5.3 kg/m^2^. The average symptom duration was 13.4 ± 8 years. Five patients had been previously treated with complete decongestive therapy (CDT) with transient good results. According to ISL staging, two patients presented with stage IIA lymphedema, four patients with stage IIB, and thirteen with stage III. Using ICG lymphography, one limb presented with a splash pattern (4.5%), seven with a stardust pattern (31.8%), and fourteen with a diffuse pattern (63.6%).

A total of 22 limbs were treated. One was treated with LVA (4.5%), one with VLNT (4.5%), eight with liposuction + LVA (36.4%), five with liposuction + VLNT (22.7%), two with RRPP + LVA (9%), two with RRPP + VLNT (9%), and three with the CHAHOVA procedure (13.6%). When performing VLNT, eight patients underwent gastroepiploic VLNT (72.7%) and three patients received supraclavicular VLNT (27.3%).

The length of hospital stay was one day for patients treated with LVA alone or combined with liposuction or RRPP, seven days for patients treated with VLNT alone or combined with liposuction or RRPP, and fourteen days for patients treated with the CHAHOVA approach. Two patients experienced minor complications: hematoma formation in one patient treated with liposuction and LVA, and a chronic ulcer in a patient treated with the CHAHOVA approach. The mean follow-up was 19 months (range: 12–24 months) (Table 1).

**Figure 3:**
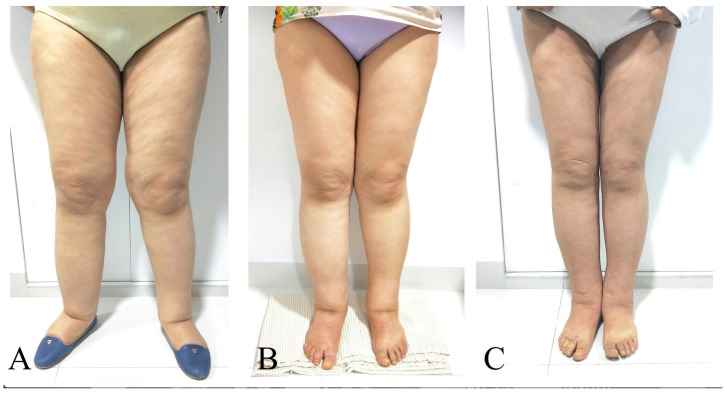
Female patient with primary bilateral lower extremity lymphedema who underwent VASER-assisted liposuction and distal LVA according to P-LYMA algorithm. (**A**) Preoperative picture. (**B**) Preoperative picture at 6 months post completion of decongestive therapy (CDT) showing improvement in the lymphatic drainage. (**C**) Postoperative picture at 24 months after surgery showing drastically significant improvement and decrease in the size of the affected limb. During the period of follow-up, the patient completed 6 months of CDT, after which it was discontinued and replaced by a single compression garment.

**Figure 4:**
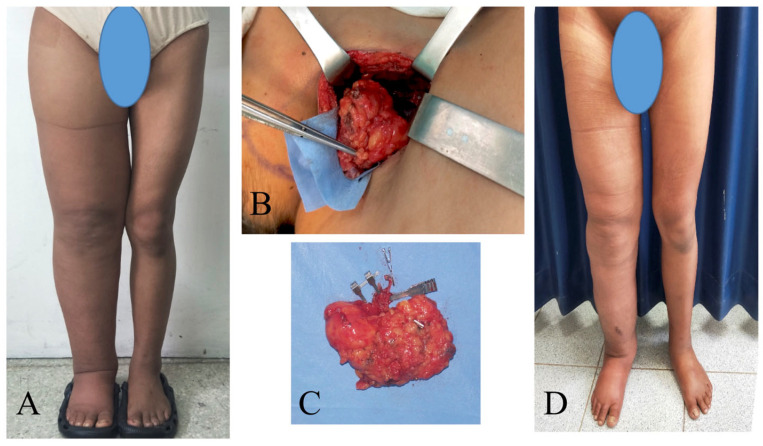
Female patient with primary right lower extremity lymphedema who underwent simultaneous VASER-assisted liposuction and supraclavicular VLNT. (**A**) Preoperative picture. (**B**) Placement of supraclavicular lymph node flap in the recipient area. (**C**) Intraoperative picture of the supraclavicular lymph node flap. (**D**) The patient completed 6 months of complete decongestive therapy and then started to wear compression garments.

The mean CRR was 65% ± 10%, 73% ± 13%, 79% ± 14%, 78% ± 17%, and 73% ± 20% at 1, 2, 3, 6, and 12 months postoperatively (Table 2). Only thirteen limbs were evaluated at 24 months with a mean reduction rate of 74% ± 29%. The episodes of cellulitis per year decreased from a mean of 1.9 ± 0.8 preoperatively to 0.4 ± 0.6 during follow-up (Figure 3 and Figure 4).

**Figure 5:**
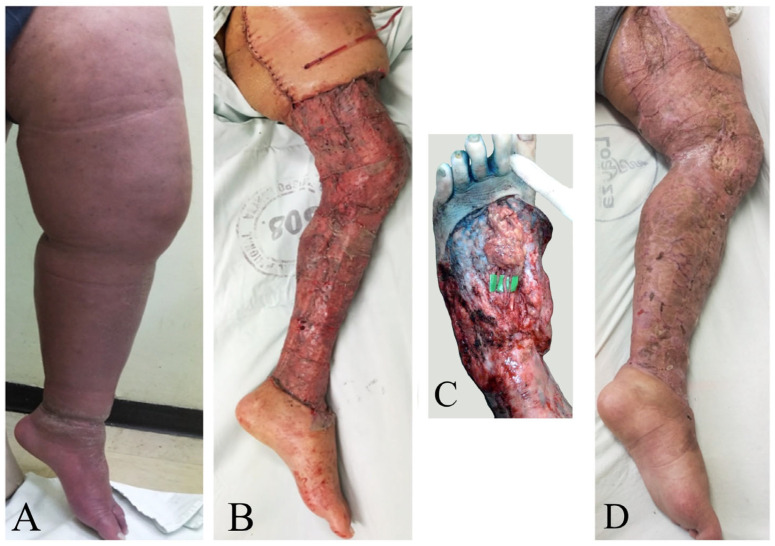
(**A**) Preoperative image showing ISL 3 lower extremity lymphedema. (**B**) Immediate postoperative photograph following Charles’ and Homan’s procedure, and VLNT (CHAHOVA). (**C**) Intraoperative picture after implantation of the gastro-epiploic lymph node flap at the foot dorsum. (**D**) Picture during follow-up at postoperative year 2 showing complete resolution of lymphedema. Complete healing and resurfacing of extremity despite chronic ulceration.

**Figure 6:**
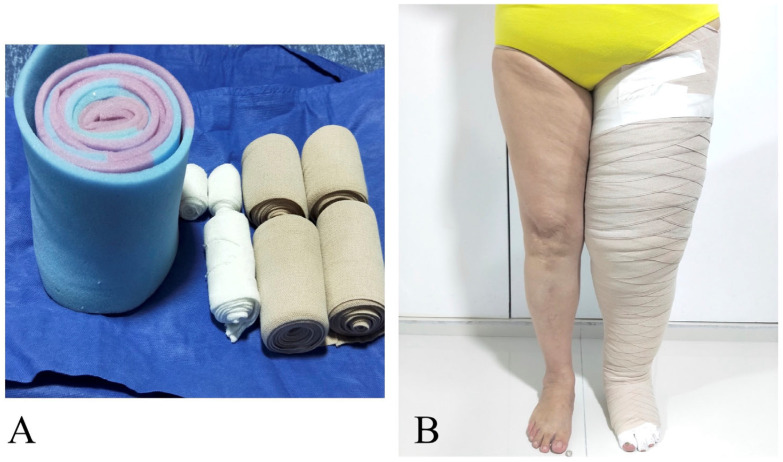
Picture shows patient with left lower extremity lymphedema using multilayer garment. This was used in all patients as part of the P-LYMA protocol during the preoperative period and 3 to 4 months postoperatively. (**A**) Materials and supplies used for multilayer bandage compression therapy. (**B**) Multilayer bandage compression therapy applied to patients left lower extremity.

**Figure 7:**
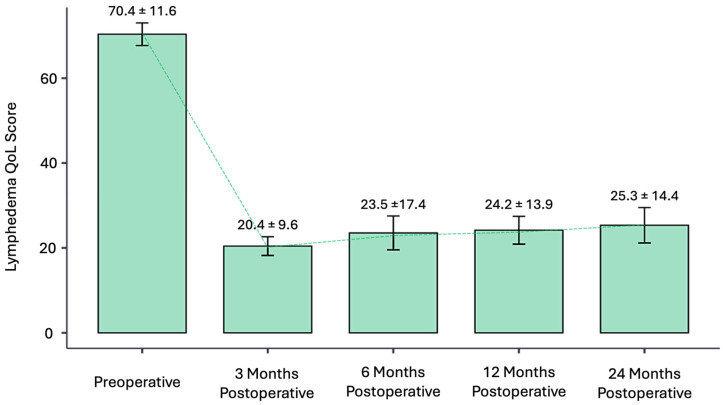
Lymphedema Quality of Life Score collected prior to surgical treatment and at 3, 6, 12, and 24 months after P-LYMA treatment. (Quality of Life, QOL).

In terms of CDT, the transition of multilayer compression therapy to traditional compression garments was performed at 3.2 months after surgery (Figure 5 and Figure 6). All patients were able to resume normal daily activities, but they all maintained the use of compression garments. Patients’ quality of life was evaluated with LeQOLiS preoperatively with a mean score of 70 ± 12. The LeQOLi score improved to 20 ± 10, 23 ± 17, and 24 ± 14 at 3, 6, and 12 months postoperatively (Figure 7). Only 12 patients had a longer follow-up of 24 months with an LeQOLi score of 25 ± 14 (Table 3).

## 4. Discussion

Primary lymphedema represents a challenge in lymphatic surgery as its pathophysiology is still poorly understood [16]. Moreover, few studies focus on primary lymphedema as it is less common than secondary lymphedema [17,18,19,20,21]. The treatment of secondary lymphedema, especially in mild cases, can still rely on a partially functional lymphatic system. Hence, the primary goal of the surgical approach is often to restore its function by locating and bypassing the disrupted areas. In primary lymphedema, however, the lymphatic system is often dysfunctional at different levels due to abnormal congenital development (i.e., aplasia, hypoplasia, and hyperplasia) and is characterized by the absence of a clear surgically correctable site [22,23]. For this reason, reconstructive approaches for primary lymphedema present numerous challenges, as the unique characteristics of the condition can significantly impact treatment effectiveness.

The present study evaluated the results of the application of an algorithm of treatment for primary lower limb lymphedema. Nineteen patients were included in this study, with a total of twenty-two limbs treated. The treatment protocol merged pre- and postoperative CDT with a combined surgical approach in order to address both the lymphatic drainage and the degeneration of the subcutaneous tissues and skin. Lymphatic drainage was improved by performing physiologic procedures (LVA or VLNT), while fibrotic adipose tissue changes were treated by reducing the burden to the limbs through excisional procedures (liposuction, RRPP, CHAHOVA).

Several authors have contrasting opinions regarding the suitability of certain physiologic procedures, such as LVA, in primary lymphedema patients. According to O’Brien, 80% of patients affected by primary lymphedema present with aplastic or hypoplastic distal superficial lymphatics. This would represent a contraindication to LVA and would limit its use to primary lymphedema patients who only have proximal obliteration of the lymphatic channels, which are only 10% of the total according to their experience [24].

On the other hand, other authors advocate that suitable lymphatics for LVA can be found in 84% of primary lymphedema patients [18]. In our experience, the wide range of different clinical conditions that can be found in patients affected by primary lymphedema strongly alters the surgical outcomes. Therefore, a complete algorithm which comprises multiple diagnostic and treatment levels is necessary to address these patients.

It is important to highlight the significance of CDT as a primary and long-lasting treatment for these patients. CDT aims to increase fluid transfer through residual functioning lymphatics, thereby reducing limb volumes and preventing disease progression caused by chronic inflammation, recurrent skin infections, and fibrosis of the subcutaneous tissues. CDT is composed of two phases: the intensive phase, aiming to reach the goals of the treatment (improve lymphatic drainage), and the maintenance phase, aiming to help preserve the current lymphodynamic properties of postoperative results [13,25,26,27]. In our study, all patients underwent CDT for six months before surgery. While CDT did not prevent surgery in our patients, it is an integral part of treatment as it helps improve the patients’ preoperative condition and prevents disease progression.

According to recent trends in lymphedema surgery, procedures performed in these patients can be divided into physiologic and excisional—with the general concept that physiologic surgery allows us to achieve better results in milder cases and earlier stages of the disease, whereas excisional procedures are more beneficial in complex and severe cases [28,29]. In our practice, we often combine a physiologic procedure with an excisional procedure in the same patient [30]. The concept behind this approach is that primary lymphedema patients are characterized by a long-lasting condition of a progressively dysfunctional lymphatic system that inevitably evolves into a chronic disorder. Therefore, in these patients, we enhance results by addressing both aspects of the disease.

Physiologic procedures are aimed at improving the system’s capability of draining the lymphatic fluid, whereas the excisional procedures allow the removal of the excess adipose and/or fibrotic tissue already formed and tissue that will not respond to physiologic treatment alone. Only in two patients with early-stage lymphedema (Stage IIA) was a single physiologic procedure performed as an excess of fat or fibrotic tissue was not identified upon clinical evaluation. The P-LYMA algorithm also aims at directing surgeons’ choice regarding the appropriate procedure to perform in each patient. However, part of the decision making is based on the experience of the surgeon.

Currently, the most commonly performed physiologic procedures are LVA and VLNT. According to the literature, the preoperative evaluation of primary lymphedema patients with lymphoscintigraphy or ICG lymphography is essential to determine which procedure would be more beneficial [31,32]. LVA has produced promising results for early-stage disease in carefully selected lymphedema patients. However, long-term results are less encouraging in advanced lymphedema stages, probably due to permanent damage from an increased interstitial pressure, recurrent infections, and lack of the functional smooth muscle required to successfully impel the lymphatic fluid into recipient veins.

Hara et al. (2015) conducted a study in which they found that preoperative ICG lymphography revealing a stardust or diffuse dermal backflow pattern in the distal aspect of the lower limb is indicative of hypoplasia in the lymphatic vessels. Based on these findings, it is suggested that in such cases, VLNT may be a more favorable treatment option compared to LVA [17]. Other studies have shown similar results in secondary lymphedema patients [33]. The P-LYMA algorithm follows this approach. Furthermore, we selected the physiologic treatment according to ICG lymphography. Patients with wide areas of dermal backflow and no suitable lymphatics were addressed with VLNT.

VLNT is a very common procedure for lymphedema patients with many studies reporting satisfying results in secondary lymphedema patients [8,34,35,36]. VLNT should be implemented in patients with minimal or no response to conservative treatment, when dermal fibrosis and sclerotic lymph vessels prevent LVA from being performed, and when postoperative outcomes following LVA are unsatisfactory. Becker et al. evaluated the effectiveness of VLNT in patients affected by primary lymphedema and reported that all cases with distal limb lymphedema experienced a reduction in the circumferences of the limbs, while in 46% of patients, the limb circumference was normalized [20]. Bolletta et al. also published an article on the adequate results of the combined approach of gastroepiploic VLNT and liposuction in patients affected by Milroy disease, a particular form of primary lymphedema [37].

The use of suction-assisted lipectomy in lymphedema patients has been evaluated in many studies and found to be effective in reducing the excess adipose tissue in the subcutaneous layer caused by adipogenesis and chronic inflammation [38,39]. In comparison to lymphedematous limbs with a predominant fluid constituent, which may be treated with physiologic procedures such as LVA and VLNT, suction-assisted lipectomy is preferably used in the cases where the solid component is predominant, as it targets the trophic changes in the subcutaneous tissue. Additionally, suction-assisted lipectomy is usually used in patients with mild fibrosis or minor trophic skin changes. This procedure, however, may not be very effective in advanced stages of disease with skin redundancy, fibrosis of the subcutaneous tissue, and recurrent infections.

A more radical approach may be required in more severe cases. One such approach is RRPP. The combination of this excisional procedure with VLNT has previously been evaluated by our senior author [11]. Advantages of RRPP when compared to liposuction include the removal of the hardened fibrotic tissue and safe redundant skin reduction by the preservation of perforators that allow us to maintain sufficient blood supply to the skin. On the other hand, chronic lymphedema can alter not only the subcutaneous tissue, which becomes fibrotic, but also the overlying skin and the underlying deep fascia [40]. The difficulties that arise in keeping the thick, papillomatous skin clean together with bacterial colonization, as well as in local immune system compromise associated with the disease, require a treatment that addresses not only limb size but also the frequent infections [41,42].

The CHAHOVA approach combines multiple procedures such as the Charles’ procedure, Homans’ procedure, and VLNT [12]. This procedure is highly invasive with frequently voiced concerns related to the aesthetic outcome. While the appearance of the limb is significantly altered by the procedure, accurate postoperative care can improve the esthetic results by reducing the rates of skin graft loss, functional joint contracture, and hypertrophic scarring. Moreover, in most of these patients, as the preoperative condition drastically reduces the function of the limb, size improvement and an improved contour of the extremity are perceived as being much more important than limb esthetics. This was confirmed in the current study, as those treated with the CHAHOVA approach reported a significant improvement in quality of life seen by the data collected with the LeQOLiS. This observation was also related to the significant reduction in the number of episodes of cellulitis which were frequent in these patients before surgery. We achieved this by treating the toes, which represented the major source of infection, as well as improving lymphatic function by transferring the lymph node flap at the distal aspect of the limb.

According to our protocol, patients are encouraged to start CDT as soon as they are diagnosed and to continue this treatment in the postoperative period. Since primary lymphedema patients are characterized by a genetically determined deficient lymphatic system, we strongly believe that the results of surgical treatment can be enhanced when combined with CDT. All our patients were evaluated throughout the follow-up and we observed that the circumference reduction rates kept improving over time. We believe this is the result of the cumulative effect of the partial lymphatic restoration and the application of decongestive therapy. In our experience, even though patients experienced an improvement as seen by the CRR and it was possible to progressively reduce the use of compression garments, we suggest that patients do not discontinue its use to maintain their results. In primary lymphedema, the lymphatic system is affected by developmental disorders, resulting in the inability of surgical procedures to completely restore the system. For this reason, the use of compression garments remains a useful tool to preserve results over time.

While a limitation of this study is the small number of patients treated, it is important to remember that primary lymphedema is less common than secondary lymphedema. Moreover, some of the patients, especially in the very early stages, are responsive to conservative treatment and are not, at least initially, addressed with surgery. To our knowledge, this is the first study that presents a complete algorithm that combines different approaches and techniques for primary lower extremity lymphedema patients. Larger multi-center studies would be needed to determine which specific methods have efficacy in certain types of the patient population. Studies with prolonged follow-up can help identify long-term outcomes and complications associated with the procedures incorporated in our algorithm.

## 5. Conclusions

The P-LYMA algorithm combines different techniques, both physiologic and excisional procedures, as well as preoperative and postoperative concomitant CDT, in order to maximize results in primary lymphedema patients and preserve them over time, thus improving patients’ quality of life in an enduring manner.

## Figures and Tables

**Table 1:** Primary lymphedema patients treated with P-LIMA algorithm: preoperative data and procedures.

Patient	Age	Gender	BMI	Affected Lower Limb	ISL Stage	ICG Pattern	Duration of Symptoms (Years)	Previous CDT	LeQOLiS	Physiologic Procedure	Excisional Procedure
1	29	F	27.5	Left	III	Diffuse	13	No	70	VLNT (SC)	SAL
2	45	F	29.4	Left	III	Diffuse	20	Irregular	86	LVA	SAL
				Right	III	Diffuse	20	Irregular		LVA	SAL
3	16	M	22.4	Right	III	Diffuse	2	No	76	LVA	SAL
4	35	F	30.0	Right	III	Stardust	28	No	82	VLNT (SC)	SAL
5	23	M	22.8	Right	II-B	Stardust	8	Irregular	85	LVA	SAL
6	16	M	24.5	Left	III	Diffuse	14	No	66	VLNT (GE)	RRPP
7	33	F	31.0	Left	III	Diffuse	30	Irregular	64	LVA	RRPP
8	15	M	19.2	Left	II-A	Stardust	2	No	66	VLNT (GE)	-
9	21	M	32.0	Left	III	Diffuse	15	No	56	VLNT (SC)	SAL
10	15	M	18.8	Right	II-B	Stardust	3	No	76	LVA	SAL
11	19	F	28.4	Left	III	Diffuse	16	Irregular	56	VLNT (GE)	SAL
				Right	III	Diffuse	15	Irregular		VLNT (GE)	SAL
12	45	M	35.2	Left	III	Diffuse	25	Irregular	52	VLNT (GE)	RRPP
13	42	M	32.4	Right	III	Diffuse	8	No	65	LVA	RRPP
14	36	M	34.4	Right	III	Diffuse	15	No	88	VLNT (GE)	CHAHOVA
15	39	F	30.4	Left	III	Diffuse	10	No	90	VLNT (GE)	CHAHOVA
				Right	III	Stardust	11	No		VLNT (GE)	CHAHOVA
16	42	M	30.2	Left	III	Stardust	12	No	66	LVA	SAL
17	23	F	24.8	Right	II-B	Stardust	5	No	68	LVA	SAL
18	37	F	32.4	Left	II-A	Splash	18	No	57	LVA	-
19	17	M	18.6	Left	II-B	Diffuse	5	No	68	LVA	SAL
**Average**	28.8		29.0				13.4		70.4		
**SD**	11.1		5.1				7.9		11.6		

Abbreviations: P-LYMA, Primary-LYmphedema Multidisciplinary Approach; BMI, body mass index; ISL, International Society of Lymphedema; ICG, indocyanine green; CDT, complex decongestive therapy; LeQOLiS, Preoperative Lymphedema Quality of Life Score; VLNT, vascularized lymph node transfer; SC, supraclavicular; GE, gastroepiploic; LVA, lymphaticovenous anastomosis; SAL, suction-assisted lipectomy; RRPP, radical reduction with preservation of perforators; CHAHOVA, combined Charles’, Homan’s, and vascularized lymph node transfer procedures.

**Table 2:** Data collected prior to surgical treatment and at 12 months and 24 months after P-LYMA treatment.

Variable	Pre-Operative	Postoperative 12 Months	Postoperative 24 Months
**Circumferential reduction rate (%)**		72.8 ± 19.6	73.9 ± 28.8
**Lymphedema Quality of Life Score**	70.4 ± 11.6	24.2 ± 13.9	25.3 ± 14.4
**Infection occurrence rate**	1.9 ± 0.8		0.4 ± 0.6

**Table 3:** Lymphedema Quality of Life Score collected prior to surgical treatment and at 3, 6, 12, and 24 months after P-LYMA treatment. (Quality of Life, QOL).

Patient	Lymphedema QoL Score Pre-Operatively	Lymphedema QoL Score Post-Op 3 Months	Lymphedema QoL Score Post-Op 6 Months	Lymphedema QoL Score Post-Op 12 Months	Lymphedema QoL Score Post-Op 24 Months
1	70	35	30	40	32
2	86	20	22	20	20
3	76	44	55	66	66
4	82	25	78		
5	85	15	8	8	8
6	66	20	22	25	22
7	64	18	10	18	
8	66	29	30	33	
9	56	10	15	18	22
10	76	6	6	8	
11	56	10	12	14	
12	52	20	25	27	
13	65	14	12	12	16
14	88	18	22	24	24
15	90	18	18	20	22
16	66	22	18	20	16
17	68	14	12	20	24
18	57	15	18	22	32
19	68	35	34	40	
**Average**	70.4 ± 11.6	20.4 ± 9.6	23.5 ±17.4	24.2 ± 13.9	25.3 ± 14.4

## Data Availability

Data are unavailable due to privacy or ethical restrictions.
